# Identification of HBEGF+ fibroblasts in the remission of rheumatoid arthritis by integrating single-cell RNA sequencing datasets and bulk RNA sequencing datasets

**DOI:** 10.1186/s13075-022-02902-x

**Published:** 2022-09-06

**Authors:** Nachun Chen, Baoying Fan, Zhiyong He, Xinping Yu, Jinjun Wang

**Affiliations:** First Department of Orthopaedics, Zhongshan City People’s Hospital Affiliated to Sun Yat-sen University, Zhongshan, Guangdong Province China

## Abstract

**Background:**

Fibroblasts are important structural cells in synovium and play key roles in maintaining the synovial homeostasis. By single-cell RNA sequencing (scRNA-seq), subpopulation of synovium-resident cells has been reported to protect intra-articular structures from chronic inflammation and promote tissue repair. However, a significant number of researchers have concentrated on the role of fibroblasts in the progress of rheumatoid arthritis (RA) while few reports had described the contribution of distinct fibroblast subsets in the RA remission. It is helpful to understand the role of fibroblast subpopulations in the RA process to provide predictive biomarkers and address RA remission mechanisms. Here, we found HBEGF+ fibroblasts that contributed to RA remission by integrating scRNA-seq datasets and bulk RNA sequencing (bulk RNA-seq) datasets.

**Method:**

Three single-cell RNA datasets of cells harvested from RA patients were processed and integrated by Seurat and Harmony R packages. After identifying cell types by classic marker genes, the integrated dataset was used to run CellChat for analysis of cell-cell communication. Specially, EGF signaling pathway was found and HBEGF+ fibroblasts were identified based on HBEGF expression. Differential expressed genes of HBEGF+ were shown in heatmap and volcano plot and used to run gene ontology (GO) enrichment analysis. Next, bulk RNA-seq datasets of synovium under different conditions (health, osteoarthritis (OA), rheumatoid arthritis, before and after classical treatment) were compared to show expression change of HBEGF and gene markers that are mainly expressed by HBEGF+ fibroblasts such as CLIC5, PDGFD, BDH2, and ENPP1. Finally, two single-cell RNA sequencing datasets of synovial cells from mice were integrated to identify Hbegf+ fibroblasts and calculate the population of Hbegf+ fibroblasts under different joint conditions (health, K/BxN serum transfer arthritis (STA), and remission of STA).

**Result:**

After integrating three single-cell RNA sequencing datasets, we identified 11 clusters of synovial cells, such as fibroblasts, mural cells, endothelial cells, CD4+ T cells, CD8+ T cells, natural killer cells, synovium macrophage, peripheral blood macrophages, plasma cells, B cells, and STMN1+ cells. We found fibroblasts had an extensive communication network with other clusters and interacted with synovial macrophages through EGF signaling pathway via analysis of cell-cell communication between synovial cells. HBEGF, ligand to EGF signaling pathway, was highly expressed by a subset of fibroblasts and macrophages, and EGFR, receptor to EGF signaling pathway, was highly expressed by fibroblasts and meniscus cells. Moreover, HBEGF was downregulated under RA state and it had an increase after classical treatment. We then defined fibroblasts with high expression of HBEGF as HBEGF+ fibroblasts. In addition, we also found that HBEGF+ fibroblasts highly expressed CRTAC1, ITGB8, SCARA5, THBS4, and ITGBL1, genes relative to encoding extracellular matrix proteins and engaged in positive regulation of cell migration and motility, cellular component movement, and cell growth by GO enrichment analysis. We eventually identified HBEGF+ fibroblasts specially expressed CLIC5, PDGFD, BDH2, and ENPP1, which positively correlated with the expression of HBEGF. Moreover, the expression of CLIC5, PDGFD, BDH2, and ENPP1 was downregulated under RA state and elevated by classical therapy. On the contrary, the HBEGF+ macrophages specially expressed SLAMF8, GK, L1RN, and JAK2, which negatively correlated with the expression of HBEGF. The expression was upregulated in SLAMF8, GK, L1RN, and JAK2 under the RA state, whereas it was decreased after classical treatment. In mice, the number of Hbegf+ fibroblasts was reduced in the RA synovium but increased in the RA remitting synovium.

**Conclusions:**

HBEGF+ fibroblasts play a role in the remission of rheumatoid arthritis, and HBEGF has potential to become a novel biomarker for prediction of RA progress.

## Introduction

Rheumatoid arthritis (RA) is a chronic and systemic autoimmune disease. It is characterized by chronic synovitis and progressive articular damage [1–3]. Accurate assessment of RA process has potential to provide optimal treatment strategies as current clinical biomarkers are unable to monitory the disease activity and a part of patients with negative inflammatory tests still have active disease [4–6]. Moreover, classification of RA synovium is possible to offer sensitive predictors of RA progress [7]. A series of studies have demonstrated that increasing numbers of mononuclear phagocytes, synovial fibroblasts, B cells, and T cells participated in RA progression and caused destruction of articular cartilage and bone [8–14]. However, few researchers paid attention to the roles of synovial cells in RA remission. Recently, with the help of single-cell transcriptome sequencing technology, tissue resident macrophages that contributed to the remission of RA were found in synovium. In human, the population of MerTK^pos^CD206^pos^ synovial tissue macrophages was higher in RA remission synovium compared to active RA synovium. Moreover, MerTK^pos^CD206^pos^ synovial tissue macrophages showed the biofunction including mediator profile resolution and repair responses [15]. In mice, locally renewing Cx3cr1+ tissue-resident macrophages form a tight-junction barrier to protect intra-articular structures from inflammatory reaction [16]. These two studies strongly indicated that there possibly may be subpopulations of synovium-resident cells with similar function in synovium and reinstating synovial homeostasis.

Fibroblasts are important structural cells in the synovium and have key roles in maintaining the homeostasis. Previous literatures have reported that fibroblasts had an important role in RA pathological processes [17]. Recent scRNA-seq analyses have also described fibroblast heterogeneity in the synovium [18] and identified specific cluster associated with RA such as THY1+ fibroblasts, which interacted with macrophages and endothelial cells and led to severe inflammatory arthritis [19, 20]. However, most of these researches concentrated on fibroblast subclasses in promoting inflammation in arthritis. Fibroblast subsets that communicate with MerTK^pos^CD206^pos^ synovial tissue macrophages and engage in tissue repair have not been reported in detail [15]. Furthermore, because of the usefulness of a detailed description of fibroblast subclasses in addressing the mechanisms of RA remission and monitoring RA activity, we hypothesized that there is a distinct population of fibroblasts producing an active role in RA remission. In this study, we integrated single-cell RNA sequencing datasets and bulk RNA-seq datasets and found HBEGF+ fibroblasts had an important role in RA remission.

## Method

### Data collection

The total of 13 datasets, including 7 single-cell transcriptomics datasets and 6 bulk RNA-seq datasets, were collected from public datasets Gene Expression Omnibus (GEO) and NIH IMMPOR (Table 1). The single-cell transcriptomics datasets contained 1 dataset of peripheral blood mononuclear cells from RA patients, 1 dataset of synovial cells from RA patients [20], 1 dataset of CD45− synovial cells from RA patients [9], 1 dataset of chondrocytes from osteoarthritis (OA) patients [21], 1 dataset of meniscus cells from OA patients [22], 1 dataset of CD45+ synovial cells mice with different states of arthritis (health and K/BxN serum transfer arthritis (STA)) [16], and 1 data of synovial cells from mice with different states of arthritis (health, STA, and STA in remission) [9]. The bulk RNA-seq datasets contained 1 dataset of fibroblasts [19] from human synovial tissue, 1 dataset of macrophages [23] from human synovial tissue, 5 multicenter datasets of synovial tissue from 102 people with different states of arthritis (health, RA, OA) [24, 25], and 1 dataset of synovial tissue from RA patients before and after drug treatment [26]. All the datasets were processed in R (V.4.0.0), and the results were showed using ggplot2 R package (V.3.3.5) except where mentioned.

**Table 1:** Induction of datasets

Accession	Tissue	RNA library	Organism	Source	Platforms
GSM4819747	PBMC from RA patient	Single-cell RNA sequencing	Homo sapiens	GEO	BGISEQ-500
SDY998	Synovial cells from RA patient	Single-cell RNA sequencing	Homo sapiens	NIH IMMPORT	Illumina HiSeq 2500
SDY1599	CD45− synovial cells from RA patient	Single-cell RNA sequencing	Homo sapiens	NIH IMMPORT	Illumina NextSeq 500
GSE104782	Chondrocytes from OA patient	Single-cell RNA sequencing	Homo sapiens	GEO	Illumina HiSeq 4000
GSE133449	Meniscus cells from OA patient	Single-cell RNA sequencing	Homo sapiens	GEO	HiSeq X Ten
GSE134420	CD45+CD11b+Ly6G− synovial cells from mice with different states of arthritis (health and K/BxN serum transfer arthritis).	Single-cell RNA sequencing	Mus musculus	GEO	Illumina HiSeq 2500
GSE145286	Synovial cells from mice with different states of arthritis (health, K/BxN serum transfer arthritis, and arthritis after treatment).	Single-cell RNA sequencing	Mus musculus	GEO	Illumina NextSeq 500
GSE77298	Synovial cells from healthy and RA patients	Bulk RNA sequencing	Homo sapiens	GEO	Affymetrix Human Genome U133 Plus 2.0 Array
GSE55584	Synovial cells from OA and RA patients	Bulk RNA sequencing	Homo sapiens	GEO	Affymetrix Human Genome U133A Array
GSE55457	Synovial cells from healthy, OA, and RA patients	Bulk RNA sequencing	Homo sapiens	GEO	Affymetrix Human Genome U133A Array
GSE55235	Synovial cells from healthy, OA, and RA patients	Bulk RNA sequencing	Homo sapiens	GEO	Affymetrix Human Genome U133A Array
GSE39340	Synovial cells from healthy, OA, and RA patients	Bulk RNA sequencing	Homo sapiens	GEO	Illumina HumanHT-12 V4.0 expression beadchip
GSE97165	Synovial tissue from RA patients before and after triple DMARD treatment	Bulk RNA sequencing	Homo sapiens	GEO	Illumina HiSeq 2000
GSE109448	Fibroblast from synovium	Bulk RNA sequencing	Homo sapiens	GEO	Illumina NextSeq 500
GSE123492	Macrophages from synovium	Bulk RNA sequencing	Homo sapiens	GEO	Illumina NextSeq 500

### Human single-cell RNA sequencing analysis

The human single-cell transcriptomics datasets, composed of 3 datasets from RA patients and 2 datasets from OA patients, were analyzed based on the states of arthritis.

#### Integrating scRNA-seq datasets of cells from RA patients

According to the Seurat single-cell analysis standard workflow [27, 28], firstly, each dataset was used to create Seurat object. Specifically, cells with <500 measured genes and >5% mitochondrial contamination were defined as low-quality cells and cells with >4500 measured genes were identified as potential doublets. They were filtered out from each dataset. After being filtered, total 29,382 cells were selected for following processes. All RA Seurat objects were merged into a different RA state object. The merged object was normalized (function NormalizeData, method = “LogNormalize,” scale. factor = 10,000), the 3000 most variable genes were identified, and the expression levels of these genes were scaled before performing PCA in variable gene space. Next, batch effect was corrected and merged object was integrated by running Harmony (version 1.0) [29]. The top 25 harmony dimensions were provided as an input for UMAP and visualized the first two UMAP dimensions at a clustering resolution of 0.2. All steps were performed using functions implemented in the Harmony package and Seurat package (NormalizeData, FindVariableFeatures, ScaleData, RunPCA, FindNeighbours, FindClusters, RunUMAP) with default parameters, except where mentioned.

Next, distinct cell types were labeled by canonical marker genes such as PRG4, PDPN (fibroblasts), THY1, MCAM (mural cells), CD34, VWF (endothelial cells), CD2, CD4 (CD4+ T cells), CD8A, GNLY, GZMB (CD8+ T cells), LTB, CD3D (natural killer cells), VSIG4, CD163 (synovium macrophages), CD68, LYZ (periperal blood macrophages), XBP1, CD27 (plasma cells), CD79A, CD37 (B cells), and STMN1 (STMN1+ cells). Gene expression of each cluster was visualized using Dotplot.

#### Integrating scRNA-seq datasets of cells from OA patients

The OA scRNA-seq datasets were integrated following the steps mentioned above with the same parameters. Total 6708 cells were involved for analysis after filtering and classified into distinct cell types referring to the source of cells. Expression of EGFR was showed using function FeaturePlot.

### Cell-cell communication in RA synovium

After identifying cell types in RA synovium, cell-cell communication was analyzed by implementing the CellChat (V.1.1.3) pipeline [30]. A new CellChat object was created from the merged Seurat object. The paracrine/autocrine signaling interaction dataset of CellChatDB was set as referencing database. Next, the communication probability was computed using a truncated mean of 20% (function computeCommunProb, type = "truncatedMean", trim = 0.2). After that, the cell-cell communication was inferred and the cell-cell communication network was aggregated with default parameters. The number of interactions was visualized to show the aggregated cell-cell communication network and signaling sent from each cell cluster. EGF signaling pathway network was showed in heatmap and ligand such as HBEGF, AREG, BTC, EGF, EREG, TGFA, and receptor EGFR, which involved in EGF signaling pathway were showed using function Featureplot based on the merged RA Seurat object.

### Bioinformatics analysis of HEBGF+ fibroblasts

When changing clustering resolution from 0.2 to 0.5 for visualizing the first two UMAP dimensions, fibroblasts that highly expressed HBEGF were divided into one group. Therefore, this group of cells was defined as HBEGF+ fibroblasts (average expression of HBEGF higher than 1.5) while fibroblasts lowly expressed HBEGF were defined as HBEGF− fibroblasts. Macrophages that highly expressed HBEGF were also defined as HBEGF+ macrophages and macrophages that lowly expressed HBEGF were defined as HBEGF− macrophages. Differential gene expression that HBEGF+ fibroblasts compared to HBEGF− fibroblasts was calculated by the function FindMarkers (Seurat R package) and showed in volcano plot. Gene expression of each cell type was computed using function FindAllMarkers (Seurat R package) and top 10 of which were showed in heatmap using function Doheatmap (Seurat R package). Expression of CLIC5, PDGFD, BDH2, ENPP1, SLAMF8, GK, L1RN, and JAK2 were displayed using function Vlnplot (Seurat R package). Differential expression gene markers of HBEGF+ fibroblasts and HBEGF− fibroblasts with the log2 fold change >1 were selected to generate a gene list for GO enrichment analysis using the clusterProfiler R package (V.4.2.2) [31, 32].

### Human bulk RNA sequencing analysis

The bulk RNA-seq datasets were consisted of 1 dataset of fibroblasts from human synovial tissue, 1 dataset of macrophages from human synovial tissue, 5 multicenter datasets of synovial tissue from 53 RA joints, 33 OA joints and 26 healthy joints, and 1 dataset of synovial tissue derived from 12 RA patients before and after classical treatment of combination of triple disease-modifying anti-rheumatic drugs (triple DMARD treatment, methotrexate, sulfasalazine, and hydroxychloroquine). Datasets from cells and synovial tissue were integrated for analysis separately. Dataset from RA joint before and after drug treatment was analyzed individually.

Datasets of fibroblasts and macrophages were merged and normalized before correcting batch effect. Sva R package (V. 3.42.0) was used to correct batch effect by running function ComBat. Then point plot was used to show the HBEGF expression in fibroblasts and macrophages. The HBEGF expression between fibroblasts and macrophages was tested by Student’s *t* test with a significance threshold of *P* < 0.05. Next, samples of fibroblasts were divided into three groups, high HBEGF group (fibroblasts with count of HBEGF higher than 1000), middle HBEGF group (fibroblasts with count of HBEGF between 100 and 1000), and low HBEGF group (fibroblasts with count of HBEGF between lower than 100). Differential gene expression analysis was performed using DESeq2 R package (V 1.36.0) between high and low HBEGF expression group and differential expression genes (log2 fold change >1 and *p* < 0.05) were showed in volcano plot.

Five multicenter bulk RNA-seq datasets were merged, normalized, and corrected following the steps mentioned above. Then the expression of HBEGF, AREG, BTC, EGF, EREG, and TGFA was showed in box plot. HBEGF and gene markers such as CLIC5, PDGFD, BDH2, ENPP1, GK, IL1B, L1RN, and SLAMF8 were tested by the Pearson correlation test and displayed in dot plot separately. Expression of HBEGF, CLIC5, PDGFD, BDH2, ENPP1, GK, IL1B, L1RN, and SLAMF8 between healthy joints and RA joints was tested by Student’s *t* test with a significance threshold of *P* < 0.05. Expression of HBEGF, CLIC5, PDGFD, BDH2, ENPP1, GK, IL1B, L1RN, and SLAMF8 before and after treatment was tested by paired Student’s *t* test and showed in box plot with a significance threshold of *P* < 0.05.

### Mouse single-cell RNA sequencing analysis

The mouse single-cell transcriptomics datasets were composed of 1 dataset of CD45+CD11b+Ly6G- synovial cells from mice with healthy joints and STA and 1 dataset of synovial cells from mice with healthy joints, STA, and STA in remission.

Two mouse scRNA-seq datasets were integrated following the steps of integrating scRNA-seq datasets of cells from RA patients with the same parameters. After filtering, total 28983 cells were involved for analysis. Next, distinct cell types were identified by marker genes such as Prg4, Pdpn, Hbegf (Hbegf+ fibroblasts and Hbegf− fibroblasts), Thy1, Mcam (mural cells), Cd34, Vwf (endothelial cells), Cd2, Cd4 (T cells), Vsig4, Cd163 (synovium macrophages), Cd79A, Cd37 (B cells), and Stmn1 (Stmn1 + cells). Expression of Hbegf was showed using the function FeaturePlot. Differential gene expression was calculated for each cell type using the function FindAllMarkers. Next, differential expression gene markers of Hbegf+ fibroblasts were ordered by the log2 fold change to generate a gene list, which was then used as an input for GSEA analysis. The top 200 differential expression gene markers of each cell type from human were used as the gene sets when running fgsea (V.1.2.0). Finally, the percentage of Hbegf+ fibroblasts was calculated under different arthritis conditions.

## Result

### Expression of HBEGF was downregulated in RA synovium but upregulated in RA remission synovium

**Fig. 1:**
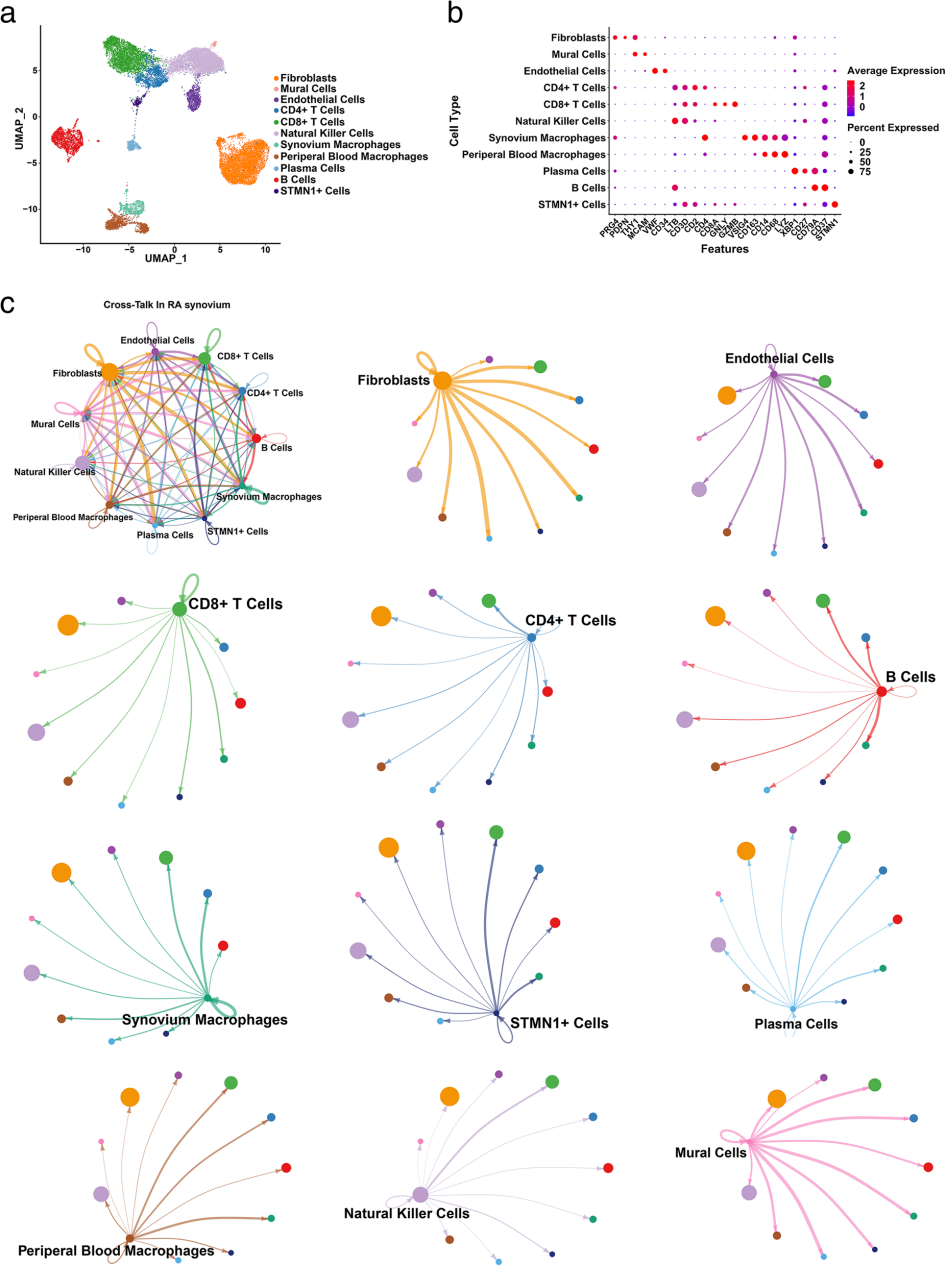
Cross-talk analysis in RA synovium. **a** UMAP of single-cell RNA-seq data of 29,382 cells from three human datasets. Eleven clusters at UMAP of integrated dataset. **b** Dot plot showing the average expression level of canonical marker genes of each cluster. **c** Cross-talk analysis between each cluster in RA synovium

A total of 29,382 cells were included in the downstream analysis after three scRNA-seq datasets had been integrated and corrected for batch effects by Harmony. Eleven clusters in RA synovium were identified and showed in uniform manifold approximation and projection (UAMP) (Fig. 1a) and defined as fibroblasts (PRG4 and PDPN), mural cells (THY1 and MCAM), endothelial cells (CD34 and VWF), CD4+ T cells (CD2 and CD4), CD8+ T cells (CD8A, GNLY, and GZMB), natural killer cells (LTB and CD3D), synovium macrophages (CD68 and LYZ), periperal blood macrophages (CD68 and LYZ), plasma cells, B cells (CD79A and CD37), and STMN1+ cells (STMN1) referring to the canonical marker genes (Fig. 1b).

Next, CellChat was employed to analyze the cell-cell communication between each cell type in the RA synovial microenvironment (Fig. 1c). We found that fibroblasts had an extensive communication network with other clusters and participated in various kinds of paracrine or autocrine signaling interactions.

**Fig. 2:**
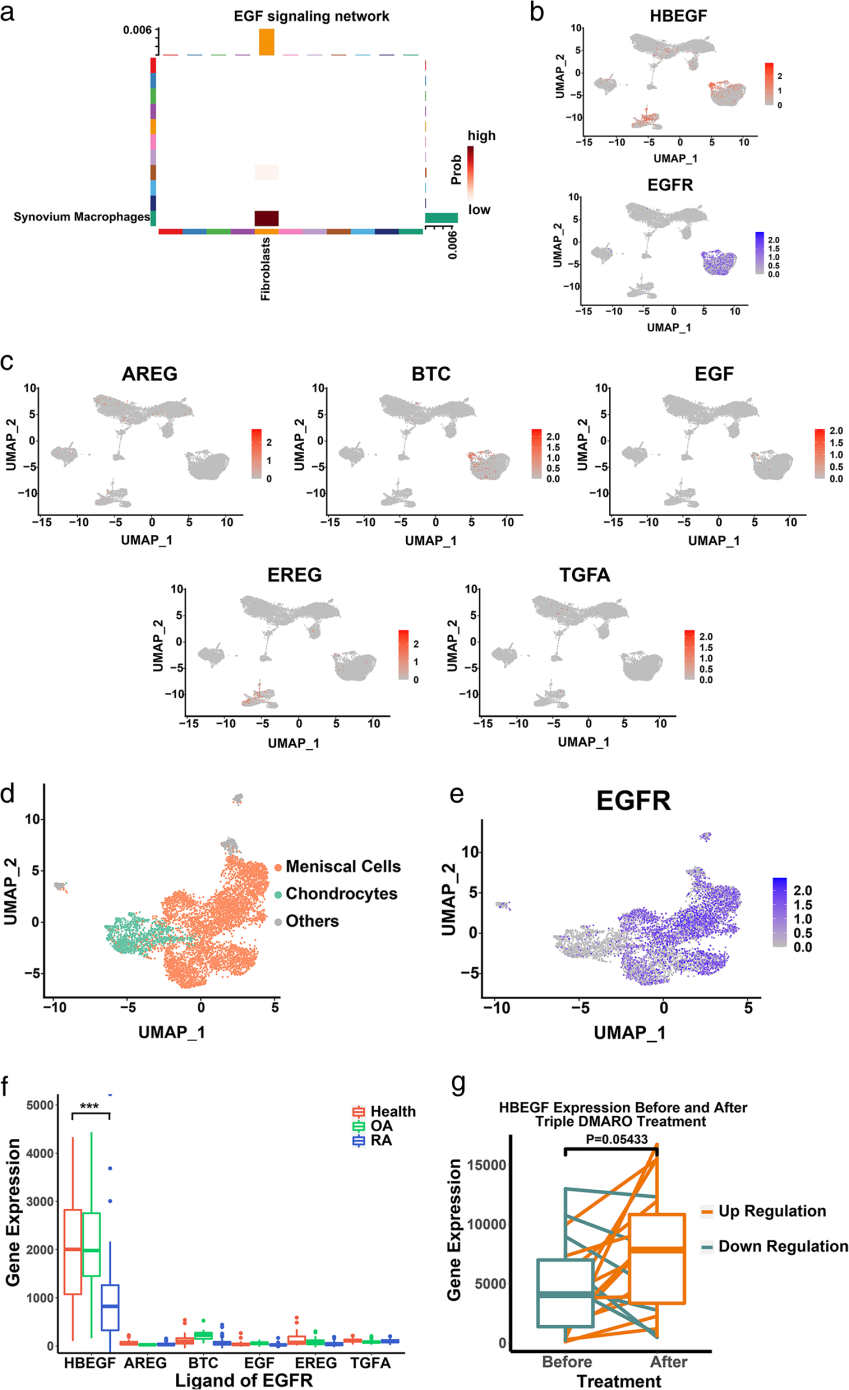
EGF signaling pathway in RA synovium. **a** Heatmap showing the EGF signaling interaction scores between each cluster in RA synovium. **b** Ligand (HBEGF) and receptor (EGFR) of EGF signaling pathway are shown in dot plots. **c** Other ligands (AREG, BTC, EGF, EREG, and TGFA) of EGF signaling pathway shown in dot plots. **d** 6708 cells from OA meniscus and cartilage at UMAP and receptor (EGFR) of EGF signaling pathway are shown in dot plot (**e**). **f** Boxplot showing expression of ligands (HBEGF, AREG, BTC, EGF, EREG, and TGFA) using bulk RNA-seq profiles of healthy joint synovium (*n* = 26), RA joint synovium (*n* = 53), and OA joint synovium (*n* = 33). Significance determined by Student’s *t* test (*P* = 0.0003). **e** Boxplot showing HBEGF expression of bulk RNA-seq profiles from RA synovium before and after triple DMARD treatment (*n*=19). Significance determined by Student’s paired *t* test (*P* = 0.05433)

Among all the signaling pathways that fibroblasts were involved, we discovered the EGF signaling pathway that was the ligand-receptor interaction between fibroblasts and synovium macrophages (Fig. 2a). HBEGF, one of the ligands in EGF signaling pathway, was mainly expressed in a part of fibroblasts and synovial macrophages (Fig. 2b). Other ligands interacting with EGFR, such as AREG, BTC, EGF, EREG, and TGFA, were lowly expressed by all clusters (Fig. 2d). EGFR, corresponding receptor to HBEGF, was only expressed by fibroblasts (Fig. 2c) in synovium. Then we noticed other tissues such as cartilage and meniscus in the joint cavity and found meniscal cells highly expressed EGFR, whereas chondrocytes did not (Fig. 2e, f). Next, we compared bulk RNA-seq data harvested from synovial cells under heathy, OA, and RA condition. HBEGF expression was significantly decreased in the RA state compared to the healthy and OA states (*P* = 0.0003, Fig. 2g). Moreover, comparing to the expression of HBEGF, expression of other EGFR ligands such as AREG, BTC, EGF, EREG, and TGFA were extremely low in all cases compared to the expression of HBEGF. After a 6-month triple DMARD treatment, the expression of HBEGF increased in RA synovium (*P* =0.05433, Fig. 2h).

### HBEGF+ fibroblast was a subset with distinct biofunction in synovium

**Fig. 3:**
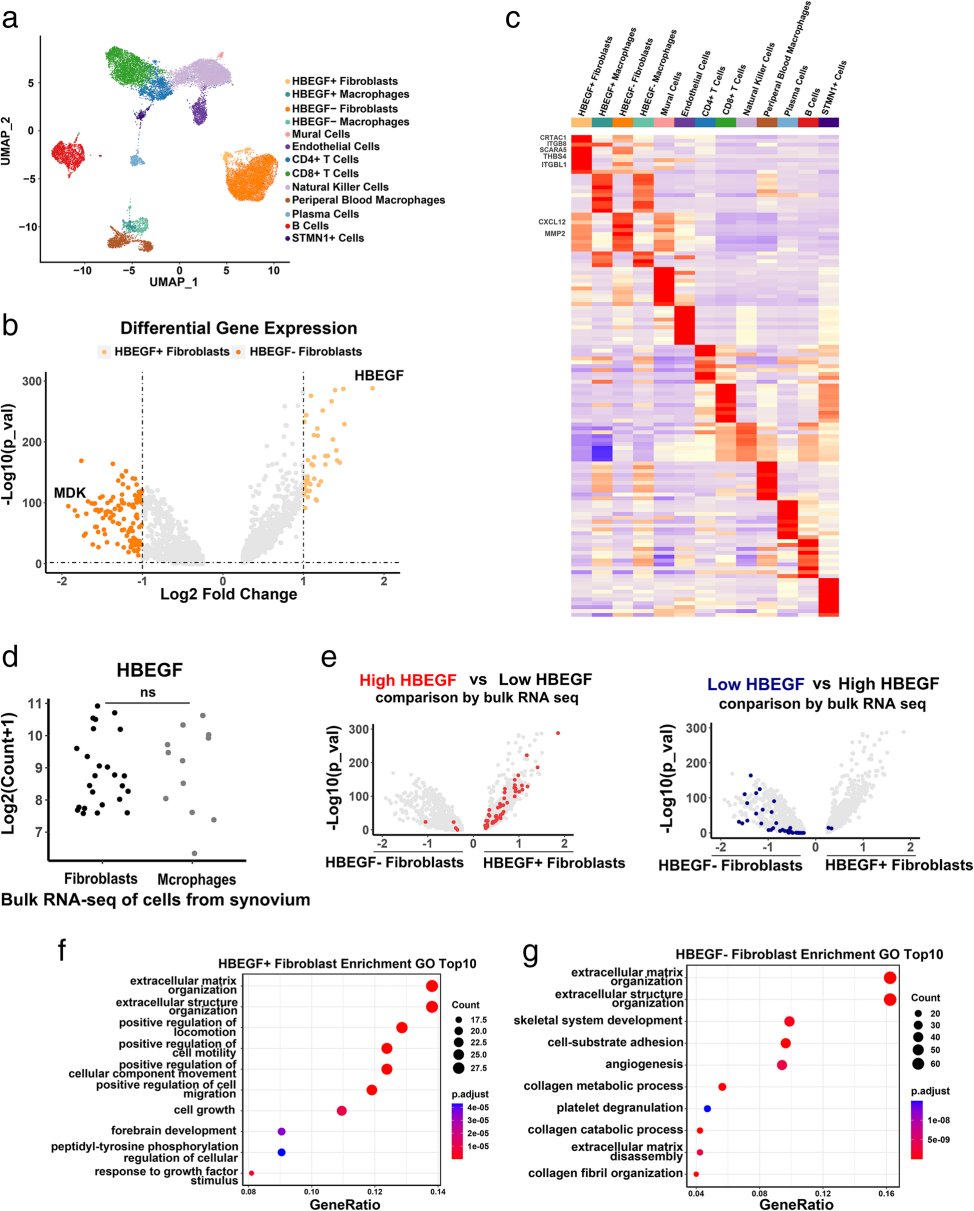
Bioinformation of HBEGF+ fibroblasts. **a** HBEGF+ fibroblasts of RA synovium are displayed at UMAP. **b** Differential gene expression of HBEGF+ fibroblasts and HBEGF− fibroblasts are shown in a volcano plot. **c** Heatmap shows top 10 expressed gene markers of each cluster. **d** HBEGF expression in synovial fibroblasts (*n* = 25) and macrophages (*n* = 12) by bulk RNA sequencing (*P* = 0.7911) are plotted as log2 count + 1. **e** Differential expression genes between high HBEGF group and low HBEGF group are highlighted on the HBEGF+ fibroblast versus HBEGF− fibroblast plot from **b**. **f**, **g** GO enrichment analysis of HBEGF+ fibroblasts (**f**) and HBEGF− fibroblasts (**g**)

As can be seen from Fig. 2b, the fibroblasts which highly expressed HBEGF (average expression of HBEGF higher than 1.5) were distributed into a small group. Then we defined them as HBEGF+ fibroblasts and fibroblasts with low expression of HBEGF were defined as HBEGF− fibroblasts. Referring to the previous studies, macrophages with high or low expression of HBEGF were defined as HBEGF+ macrophages and HBEGF− macrophages separately (Fig. 3a).

By calculating the differential gene expression between HBEGF+ fibroblasts and HBEGF− fibroblasts, we detected 162 genes with distinct expression patterns between HBEGF+ fibroblasts and HBEGF− fibroblasts. Forty-four gene markers were highly expressed by HBEGF+ fibroblasts and 118 gene markers by HBEGF− fibroblasts. The main differential expressed gene of HBEGF+ fibroblasts was HBEGF whereas the differentially expressed gene in HBEGF− fibroblasts is MDK, which is related to the pathogenesis of RA (Fig. 3b). Next, we figured out genes mainly expressing by each cluster and found HEBGF+ fibroblasts were heterogeneous from other subpopulations with high expression of CRTAC1, ITGB8, SCARA5, THBS4, and ITGBL1 while HBEGF− fibroblasts highly expressed CXCL12 and MMP2 (Fig. 3c). In bulk RNA sequencing datasets of synovial fibroblasts and macrophages, we also found subsets of fibroblasts and macrophages highly expressing HBEGF in synovium (Fig. 3d). Comparing to low HBEGF group (fibroblasts with count of HBEGF lower than 100), we noted that the differential expression genes (DEG) of high HBEGF group (fibroblast with count of HBEGF higher than 1000) were more abundant in HBEGF+ fibroblasts.

Finally, we used GO enrichment analysis to figure out the difference between HBEGF+ fibroblasts and HBEGF− fibroblasts in biofunction. We found that HBEGF+ fibroblasts involved in positive regulation of cell migration and motility, cellular component movement and cell growth whereas HBEGF− fibroblasts engaged in collagen metabolic and catabolic process and angiogenesis (Fig. 3f, g).

### Population of HBEGF+ fibroblasts was decreased in RA synovium but increased in RA remission synovium

**Fig. 4:**
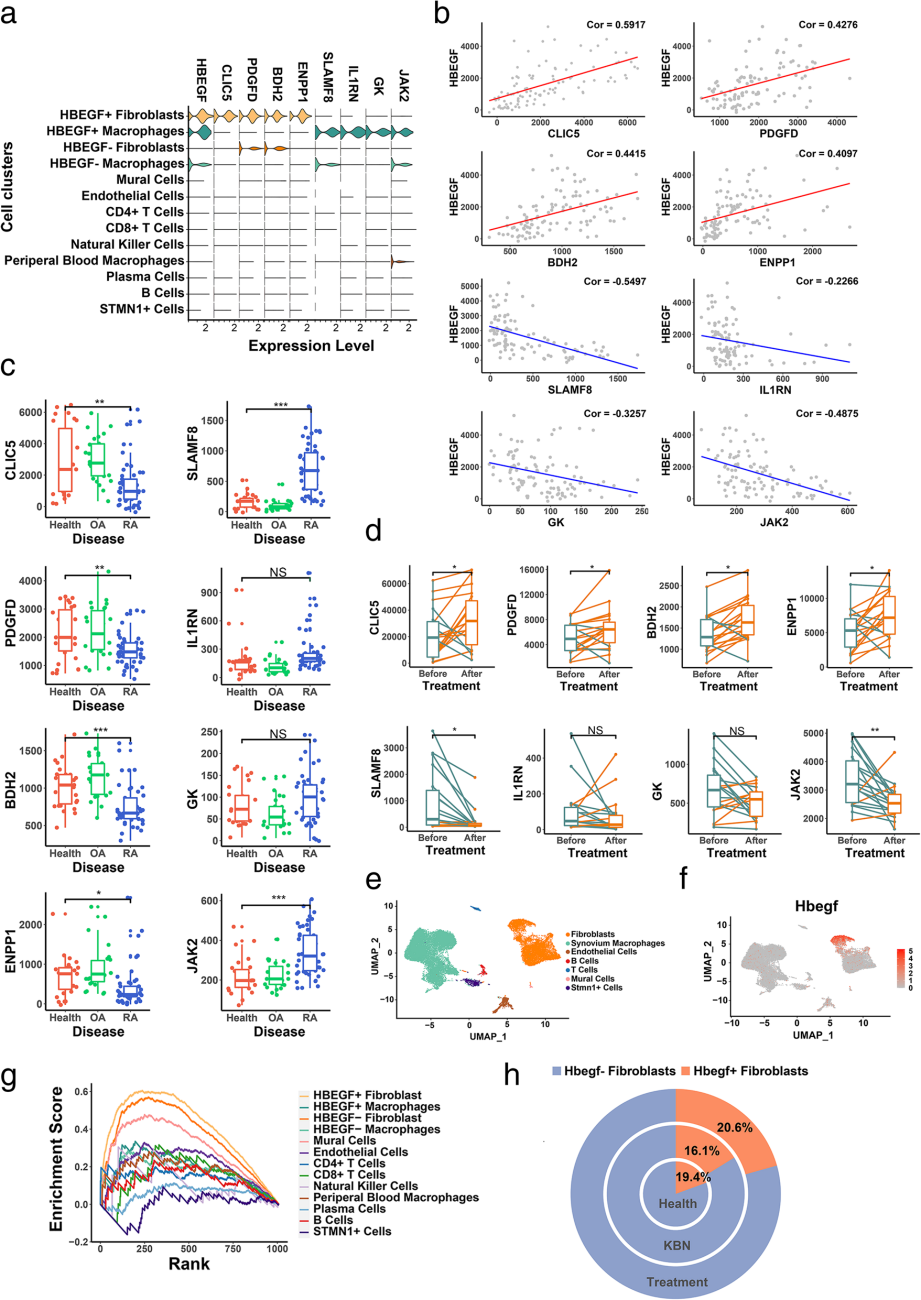
Population change of HBEGF+ fibroblasts. **a** Expression of CLIC5, PDGFD, BDH2, ENPP1, SLAMF8, IL1RN, GK, and JAK2 are shown in Vin plot using single-cell RNA-seq profiles. **b** The Pearson correlation between HBEGF and gene markers such as CLIC5 (Cor = 0.5917), PDGFD (Cor = 0.4276), BDH2 (Cor = 0.4415), ENPP1 (Cor = 0.4097), SLAMF8 (Cor = −0.5497), L1RN (Cor = −0.2266), GK (Cor = −0.3257), and JAK2 (Cor = −0.4875) is displayed on a dot plot. **c** Expression of CLIC5, PDGFD, BDH2, ENPP1, SLAMF8, IL1RN, GK, and JAK2 are displayed in boxplot using bulk RNA-seq profiles of healthy joint synovium (*n* =26), RA joint synovium (*n* = 53), and OA joint synovium (*n* = 33). Significance determined by Student’s *t* test (CLIC5, *P* = 0.0018, PDGFD, *P* = 0.0238, BDH2, *P* = 0.0001, ENPP1, *P* = 0.0399, SLAMF8, *P* = 0.0001, L1RN, *P* = 0.0602, GK, *P* =0.1461, JAK2, *P* = 0.0001). **d** Expression of CLIC5, PDGFD, BDH2, ENPP1, SLAMF8, IL1RN, GK, and JAK2 displayed in a boxplot using bulk RNA-seq profiles from RA synovium before and after triple DMARD treatment (*n*=19). Significance determined by Student’s paired *t* test (CLIC5, *P* = 0.0355, PDGFD, *P* = 0.0022, BDH2, *P* = 0.0010, ENPP1, *P* = 0.0124, SLAMF8, *P* = 0.0038, L1RN, *P* = 0.4620, GK, *P* =0.1461, JAK2, *P* = 0.0048). **e** UMAP projection of single-cell RNA-seq data of 28,983 cells from two mouse datasets. Seven clusters at UMAP of integrated dataset. **f** Expression of Hbegf shown in dot plot. **g** GSEA using top 200 expressed gene markers of each cell cluster in human synovium as gene sets and ranked gene lists from Hbegf+ fibroblasts in mouse synovium. Normalized enrichment scores of HBEGF+ fibroblasts, NES = 1.6723, *P*.adj = 0.0001. **h** Percentage of Hbegf+ fibroblasts in synovium under different states (health, STA, and STA after treatment)

To evaluate the population changes of HBEGF+ fibroblasts in different states of synovium, we investigated gene markers that are specially expressed by HBEGF+ fibroblasts and HBEGF+ macrophages. We found that CLIC5, PDGFD, BDH2, and ENPP1 were mainly expressed by HBEGF+ fibroblasts, and SLAMF8, GK, L1RN, and JAK2 were mainly expressed by HBEGF+ macrophages (Fig. 4a).

Then, we selected these gene markers for the Pearson correlation test in the integrated bulk RNA sequencing dataset and found that the expression of CLIC5, PDGFD, BDH2, and ENPP1 was positively correlated with expression of HBEGF while the expression of SLAMF8, GK, L1RN, and JAK2 was negatively correlated with expression of HBEGF (Fig. 4b). Expression of CLIC5, PDGFD, BDH2, and ENPP1 was decreased, while expression of SLAMF8, GK, L1RN, and JAK2 was increased compared to synovial membranes in healthy and OA states (Fig. 4c). Moreover, after triple DMARD treatment, the expression of CLIC5, PDGFD, BDH2, and ENPP1 had an increase in most of patients. Conversely, the expression of SLAMF8, GK, L1RN, and JAK2 had a decrease after treatment in most of patients (Fig. 4d).

Next, we analyzed single-cell RNA sequencing datasets from mouse with different states of arthritis (healthy, STA, and STA after treatment). Total 28,983 cells included fibroblasts, mural cell, endothelial cells, T cells, synovium macrophages, B cells, and STMN1+ cells (Fig. 4e). Hbegf+ fibroblasts also existed in mouse synovium (Fig. 4f). However, few macrophages with high expression of Hbegf could be found in mouse synovium. To prove that Hbegf+ fibroblasts from mice were similar to HBEGF+ fibroblasts from human, we ran GSEA analysis and found the phenotype of Hbegf+ fibroblasts from mice aligned closer to HBEGF+ fibroblasts than the other clusters from human (Fig. 4g). Finally, we found that the population change of Hbegf+ fibroblasts had a similar pattern as the expression change of HBEGF under different conditions. The proportion of Hbegf+ fibroblasts decreased in RA synovium comparing to healthy tissue. However, after treatment, the proportion of Hbegf+ fibroblasts returned to a healthy level (Fig. 4h).

## Discussion

In rheumatoid arthritis (RA), synovial fibroblasts have been considered as the key roles in regulation of joint homeostasis [10, 12, 14, 17–20]. Corresponding with previous researches, our study confirmed that fibroblasts had an extensive communication network with other clusters in RA synovium. Among the cell-cell communications between fibroblasts and other clusters, we found EGF singling pathway and HBEGF+ fibroblasts.

This study subdivided fibroblasts into 2 clusters based on the expression of HBEGF. Fibroblasts that highly expressed HBEGF (average expression of HBEGF higher than 1.5) were defined as HBEGF+ fibroblasts while the population with low expression of HBEGF were regarded as HBEGF− fibroblast. HBEGF, heparin-binding EGF-like growth factor, is one of the ligands for the ErbB family of epidermal growth factor receptors (including EGFR) [33]. It stimulates the migration, differentiation, and proliferation of cells to fill the damaged area and repair tissue lesion. Recent literatures have reported the protective function of HBEGF in TNF-driven chronic intestinal inflammation [34] and cartilage degeneration diseases [35]. And this study demonstrated HBEGF expression was downregulated in RA synovium and increased after classic therapeutic strategy—triple DMARD treatment. A similar pattern could be seen in the population of Hbegf+ fibroblasts in mice with different states of arthritis. The amount of Hbegf+ fibroblasts decreased in RA joints but increased in RA remission joints. And GO enrichment analysis showed that HBEGF+ fibroblasts played a role in cell growth and positive regulation of cell migration and motility cellular component movement while HBEGF− fibroblasts showed opposite biofunction and involved in collagen metabolic process and angiogenesis which were proven to promote inflammation in arthritis [36–38]. Therefore, we believe that HBEGF+ fibroblasts played essential roles in the remission of RA. And we also believe that further researches on HBEGF+ fibroblasts could help to address the mechanism of RA remission and may identify novel biomarkers for the prediction of RA process.

In synovium, a fraction of synovial macrophages also highly expressed HBEGF. Previous research defined them as HBEGF+ macrophages and described their function in RA synovium [9]. HBEGF+ macrophages activated synovial fibroblasts and subsequently induced invasiveness in synovium. In order to figure out the primary source of HBEGF, we used the Pearson correlation test and found that expression of HBEGF was more relative to HBEGF+ fibroblasts than HBEGF+ macrophages. In mice, we could find Hbegf+ fibroblasts exist in synovium but Hbegf+ macrophages did not. Furthermore, the population change of Hbegf+ fibroblasts showed the similar pattern as the expression change of HBEGF. The population of Hbegf+ fibroblasts decreased in RA state synovium and had an increase after RA remission. That meant HBEGF+ fibroblasts, instead of HBEGF+ macrophages, were the primary source of HBEGF in synovium and the population change of HBEGF+ fibroblasts was a reason for the expression change of HBEGF.

This study has proven that expression of HBEGF was downregulated in RA state synovium. However, pervious literatures had pointed out that the importance of the ErbB family pathway in chronic pain and activating EGF singling pathway resulted in deterioration of RA. For instance, injecting HBEGF into the paw of mice caused painful mechanical hypersensitivity and severe pain and blocking the ErbB receptor could alleviate RA pain and joint inflammation [39, 40]. In vivo, there are two different structural forms of HBEGF including proHBEGF (transmembrane protein) and sHBEGF (soluble protein) [41, 42]. proHBEGF is a precursor for sHBEGF and can be cleaved at the plasma membrane to yield sHBEGF. proHBEGF takes part in juxtacrine activity and sHBEGF engages in paracrine activity. From scRNA-seq data, we noticed that HBEGF+ fibroblasts accounted for a small part comparing to the huge amount of HBEGF− fibroblasts. And specially, the ErbB receptor, EGFR, was highly expressed by HBEGF− fibroblasts, which also highly expressed MDK (Midkine) [43], and CXCL12 (C-X-C Motif Chemokine Ligand 12) [44, 45] that engage in the pathophysiology of RA. So it is possible that the different spatial location between HEBGF+ fibroblasts and HEBGF− fibroblasts in some ways prevents proHBEGF from contacting with EGFR and activating HBEGF− fibroblasts. Moreover, small quantities of HBEGF+ fibroblasts limit the production of sHBEGF and in other ways inhibit the interaction of sHBEGF with HEBGF− fibroblasts. Oppositely, injecting exogenous HBEGF, which served as sHBEGF, mediated EGFR expressed by HBEGF− fibroblasts directly and subsequently activated HBEGF− fibroblasts to secrete increasingly cytokines and chemokines.

## Conclusions

In summary, population of HBEGF+ fibroblasts and expression of HBEGF decreased in RA synovium and increased after treatment. To conclude, HBEGF+ fibroblasts play a role in the remission of rheumatoid arthritis and HBEGF has potential to become a novel biomarker for prediction of RA progress.

## Data Availability

All the data of this manuscript was collected from public datasets such as GEO and NIH IMMPOR.

## Abbreviations

HBEGF: Heparin-binding EGF-like growth factor
scRNA-seq: Single-cell RNA sequencing
RA: Rheumatoid arthritis
OA: Osteoarthritis
GO enrichment analysis: Gene ontology enrichment analysis
UAMP: Uniform manifold approximation and projection
EGF: Epidermal growth factor receptor
EGFR: Epidermal growth factor receptor
GEO: Gene Expression Omnibus
DEG: Differential expression genes
triple DMARD treatment: Combination of triple disease-modifying anti-rheumatic drugs
STA: K/BxN serum transfer arthritis
MDK: Midkine
CXCL12: C-X-C Motif Chemokine Ligand 12
